# Acupuncture and Related Therapies for Chronic Urticaria: A Critical Overview of Systematic Reviews

**DOI:** 10.1155/2022/2094589

**Published:** 2022-10-27

**Authors:** Yun-Zhou Shi, Wei Cao, Chun-Xiao Li, Xian-Jun Xiao, Ying Huang, Lei-Xiao Zhang, Zi-Hao Zou, Si-Jue Chen, Qian Yang, Lu Wang, Ping-Sheng Hao, Zi-Ping Gao, Ying Li

**Affiliations:** ^1^Department of Acupuncture and Moxibustion, Chengdu University of Traditional Chinese Medicine, Chengdu, China; ^2^The Dermatology Department of the Affiliated Hospital of Chengdu University of Traditional Chinese Medicine, Chengdu, China; ^3^Department of Integrated Traditional and Western Medicine, West China Hospital, Sichuan University, Chengdu, China; ^4^Acupuncture and Moxibustion School of Health Preservation and Rehabilitation, Chengdu University of Traditional Chinese Medicine, Chengdu, China

## Abstract

**Background:**

Chronic urticaria (CU) can severely impair the quality of life. Acupuncture and related therapies have been widely used in the treatment of CU in China. This study aimed to summarize and critically evaluate the methodological and reporting quality of relevant systematic reviews (SRs) and present objective and comprehensive evidence on the effectiveness and safety of acupuncture and related therapies for CU.

**Methods:**

Eight electronic databases were searched from inception to October 2021 for SRs examining acupuncture and related therapies for CU, and gray literature was manually searched. Two authors independently identified SRs and extracted data. The methodological and reporting quality of these SRs were assessed by the Assessment of Multiple Systematic Reviews 2 (AMSTAR 2) tool and preferred reporting items for SRs and meta-analyses (PRISMA, 2020), respectively. In addition, the risk of bias in systematic reviews (ROBIS) was used to evaluate the risk of bias. The Grading of Recommendations Assessment, Development, and Evaluation (GRADE) framework was applied to evaluate the quality of evidence for outcome measures.

**Results:**

In total, 23 SRs, including a total of 11 outcome indicators, were published before October 2021. The AMSTAR-2 results showed that the methodological quality of all SRs was critically low; items 2, 3, 7, 9, 10, and 16 were found to have particularly low quality. For PRISMA, the reporting quality of the included SRs was unsatisfactory, and major reporting flaws were observed in the search strategy, synthesis method, certainly assessment, reporting biases, registrations, and financial support of the included SRs. For ROBIS, 22 SRs (95.65%) had a high risk of bias. Among the 55 outcomes assessed using the GRADE framework, there were 3 (5.45%) outcomes with moderate-quality evidence, 6 (10.91%) outcomes with low-quality evidence, and 46 (83.64%) outcomes with very low-quality evidence. We found the moderate quality of evidence indicating that the total effective rate and curing rate of the acupuncture group were higher than those of the western medicine group, and the recurrence rate was lower than that of the western medicine group.

**Conclusions:**

Acupuncture and related therapies for the treatment of CU are supported by low-quality evidence-based medicine. However, considering the poor quality of these SRs, we suggest that studies with more rigorous designs, larger sample sizes, and higher methodological and reporting quality are necessary to provide stronger evidence. *Registration*. The protocol for this study has been registered (PROSPERO registration number: CRD42021259131).

## 1. Introduction

Urticaria is a skin disease marked by the appearance of wheals (hives), angioedema, or both [1, 2]. When a person has transient wheals that last more than 6 weeks and occur virtually daily [2, 3], it is defined as chronic urticaria (CU). The CU can affect approximately 1% of the world's population of all ages, mainly young and middle-aged women [4]. CU can cause anxiety, depression, sleep and sexuality disturbances, and severely impair quality of life [5–8]. According to the evidence, patients with CSU can experience a significant loss of productivity at work, school, or in daily activities [9, 10]. In addition, the decline in productivity also leads to high direct and indirect healthcare costs to treat CU, with large socioeconomic impacts [4, 11, 12].

The current international guidelines [1] and consensus statements [3, 13] highly suggest a stepwise approach to complete symptom control. However, many patients have an inadequate response to the majority of these drugs [14]. Furthermore, if patients continue to be symptomatic despite the use of H1-antihistamines, the guidelines do not provide guidance on the selection, dose, and duration of alternative treatment options. Although omalizumab and CsA have been shown to be effective [15–17], the prices are high and can place a significant financial burden on patients. Their widespread adoption will be determined by legal and economic factors [18]. As a result, an increasing number of patients are seeking alternative treatment options.

In China, acupuncture and related therapies have been widely used to treat CU. At present, a series of systematic reviews (SRs) have proven the efficacy and safety of specific treatments [19–41]. In the field of evidence-based medicine, SRs have long been regarded as one of the most important sources of high-quality, trustworthy information. However, these SRs examining acupuncture and related therapies for CU were published in different years, included various acupuncture methods, and examined different outcomes. The conclusions are not completely consistent among these SRs. Therefore, a rigorous methodological approach is used herein to summarize and critically assess the methodological and reporting quality of relevant SRs, as well as to provide objective and comprehensive findings on the efficacy and safety of acupuncture and related therapies for CU. We believe this work can help to provide a better reference and evidence support for acupuncture and related therapies in CU treatment.

## 2. Materials and Methods

### 2.1. Search Strategy

We searched the following electronic databases from inception to October 2021: PubMed, Embase, Web of Science, Cochrane Library, Chinese Biological Medicine Database (CBM), Chinese National Knowledge Infrastructure (CNKI), China Science and Technology Journal Database (VIP), and Wang Fang Data Database. The search strategy included keywords and medical subject headings for “acupuncture,” “urticaria,” “systematic review,” and other related terms. In addition, additional citations were discovered by manual searches of reference lists and personal collections.  Supplemental 1 includes the detailed search strategy for the eight electronic databases mentioned above.

### 2.2. Study Selection

Two reviewers independently identified studies that met the following criteria: (i) randomized controlled trials (RCTs), quasi-randomized control trials, or controlled clinical trials (CCTs) were included in SRs; (ii) all interventional SRs that investigated the efficacy and safety of acupuncture and related therapies in CU were eligible. There were no restrictions based on age, gender, nationality, or geographic location; (iii) either acupuncture and related therapies alone or combined with other conventional Western medicine were recognized as a treatment intervention; the control types included conventional treatments that were different from the treatment intervention, placebo, or no treatment; (iv) at least one of the following outcome measures listed below was required to be obtainable: total efficiency, adverse reaction rate, recurrence rate, clinical efficacy rate, improvement rate, curing rate, IgE levels, Dermatology Quality Life Index (DLQI), quality of life score (QoL), a total improvement of clinical signs and symptoms, disease activity control, and Itch Severity Score (ISS); (v) SRs published in English or Chinese were included. SRs that were noninterventional SRs, comments, guidelines, editorials, incomplete articles, proceedings, and responses were excluded. Disagreements were settled through discussion and consensus.

### 2.3. Data Abstraction

One reviewer extracted descriptive data using a standardized form, which was then verified by a second reviewer. The following data were extracted: author, published country, age, study type, number of trials included, interventional methods, comparator, outcomes, methods for primary study quality assessment, etc. Based on what the review authors reported, data from these SRs were expressed as standardized mean differences, weighted mean differences, odds ratios (ORs), or relative risks (RRs). Whenever possible, the results of the meta-analysis are also presented with 95% confidence intervals (CIs).

### 2.4. Quality of Included SRs

#### 2.4.1. Methodological Quality of Included SRs

Two reviewers independently assessed the methodological quality of these SRs using the Assessment of Multiple Systematic Reviews 2 (AMSTAR 2) tool. The AMSTAR has a strong face and content validity for measuring the methodological quality of SRs as a methodological quality evaluation tool [42, 43]. In 2017, the expert group of AMSTAR reported on the updating and adaptation of AMSTAR [44] to allow for a more detailed assessment of SRs that includes randomized or nonrandomized studies of healthcare interventions, or both. There are 16 items in the AMSTAR 2 tool. If the item explanation was satisfied, the judgment was “Y”; if the item was answered correctly with limited information, it was evaluated as “Partial Yes”; and if the item was not subject to relevant evaluation or was evaluated incorrectly, it was evaluated as “No”. The ratings of overall confidence on the AMSTAR2 scale ranged from high to critically low. The presence of more than one critical flaw, with or without noncritical flaws, indicated critically low; one critical flaw, with or without noncritical weaknesses, indicated low quality; more than one noncritical weakness indicated moderate quality; and no or one noncritical weakness indicated high quality.

#### 2.4.2. Reporting Quality of Included SRs

Two reviewers independently evaluated the reporting quality of the included SRs using PRISMA 2020 checklists [45, 46]. The PRISMA 2020 checklist consists of 27 items and includes items deemed essential for the transparent reporting of an SR. It was evaluated as “Yes” if the item was answered as completely correct and well documented; it was evaluated as “Partial Yes” if the item was answered correctly with limited evidence; it was evaluated as “No” if the item was not subjected to relevant evaluation or was evaluated incorrectly.

#### 2.4.3. Risk of Bias of Included SRs

Using the risk of bias in systematic reviews (ROBIS) tool, two reviewers independently assessed the risk of bias of the included SRs [47]. The ROBIS tool contains the following 2 phases with 4 domains: “study eligibility criteria,” “identification and selection of studies,” “data collection and study appraisal,” and “synthesis and findings.” Each domain contains signaling questions as well as a judgment of concerns about the domain's risk of bias, and the results are regarded as “high risk,” “low risk,” or “unclear risk.”

#### 2.4.4. Evidence Quality of Included SRs

The GRADE (Grading of Recommendations Assessment, Development, and Evaluation) was used to assess the evidence quality of the included SRs [48]. GRADE pro 3.2 software includes five downgrading factors (bias risk, inconsistency, indirectness, imprecision, and publication bias) and three upgrading factors (a large magnitude of the effect, the influence of all plausible residual confounding, and the dose-response gradient). In this study, only SRs included in RCTs are included, so only 5 downgrading factors need to be analyzed. Among them, inconsistency was mainly divided into no (I^2^ < 50%), serious (50% < I^2^ < 80%), and very serious (I^2^ > 80%). Imprecision determined whether the total sample size met the optimal information size, and publication bias was determined according to the funnel plot. Each outcome of the included studies was evaluated by two reviewers. Disagreements were settled through consensus or by consulting a third investigator.

All included SRs were evaluated by two independent reviewers. We used SPSS 23.0 statistical software (IBM Corp., Armonk, New York, USA) for consistency analysis, and the kappa consistency test was used to evaluate [49]. Inconsistent results were reevaluated by another reviewer.

## 3. Results

**Figure 1:**
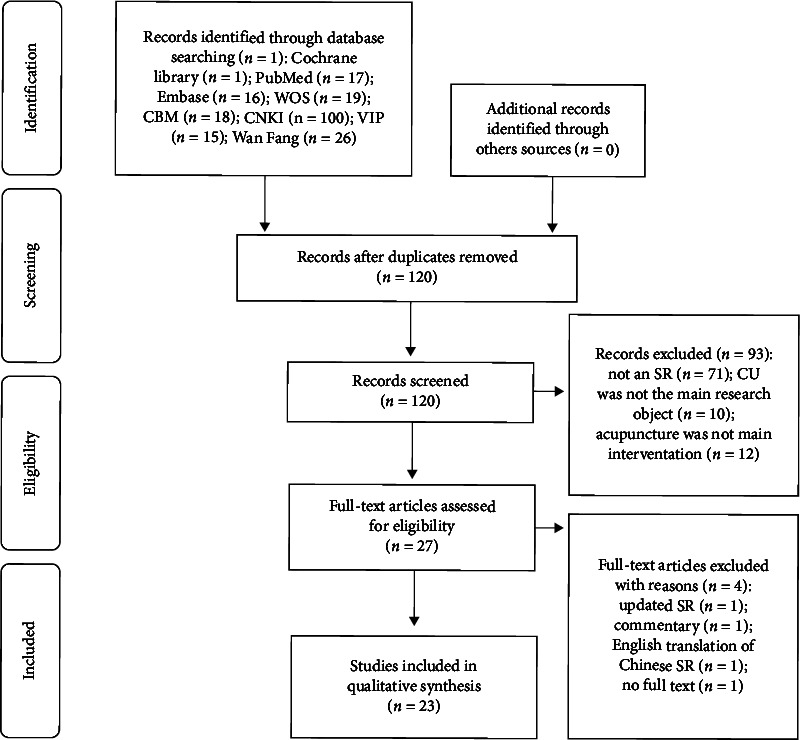
The flow of studies through the selection.

A total of 212 articles were detected in the initial search. 92 duplicate articles were excluded by using EndNote and manual searches. After screening the titles and abstracts, 93 articles were excluded. Then, full texts were screened according to the inclusion and exclusion criteria, and 4 articles were excluded. Ultimately, 23 SRs were included in the present study.  Figure 1 presents the flow of studies through the selection process.

### 3.1. Basic Characteristics of the Included SRs

The 23 included SRs were published between 2009 and 2021. Among the 23 SRs, 5 English papers and 18 Chinese papers were included, and the number of RCTs examined in the SRs ranged from 5 to 16. All reviews were conducted in China, and a total of 11 outcome indicators were reported. In terms of intervention measures, among the 23 SRs, 10 SRs examined autohemotherapy at acupoints [28–30, 33, 35–39, 41], 6 SRs examined acupuncture [22, 24, 25, 27, 34, 40], 3 SRs examined catgut embedding at acupoints [26, 31, 32], 1 SR examined bloodletting therapy [20], 1 SR examined auriculotherapy [21], 1 SR examined cupping [23], and 1 SR examined acupoint stimulation [19]. For the assessment of methodological quality, 17 SRs used the Cochrane risk of bias tool [19–23, 25–29, 31, 33, 36, 37, 39–41], 4 SRs used the Jadad scale [32, 34, 35, 38], and 2 SRs used the Jadad scale and Cochrane risk of bias tool [24, 30]. All SRs included a meta-analysis; 9 out of 23 SRs included subgroup analysis [19, 23, 24, 29, 30, 38–41]; and 13 SRs included sensitivity analysis [20, 21, 25–27, 29–32, 37–40]. The general characteristics of these SRs are summarized in Table 1.

### 3.2. Quality of Included SRs

#### 3.2.1. Methodological Quality of Included SRs

Table 2 shows the rating overall confidence of the individual quality components based on the AMSTAR 2 tool. All SRs were of critically low quality. Items 2, 3, 7, 9, 10, and 16 were rated as particularly low quality. Among the 23 SRs, twenty (86.96%) SRs used the Patient Interventions Control Outcomes (PICO) description as an organizing framework for a study question and used a satisfactory technique for assessing the risk of bias (RoB). All review authors applied appropriate methods for statistical synthesis. However, only a small proportion (8.70%) of these SRs provided the protocol or registered information [22, 23]. No SR explained the selective inclusion of the study designs and assessed the potential impact of RoB in individual studies on the results of the meta-analysis. In addition, no SR provided a list of excluded studies and justified the exclusions. Only 2 (8.70%) SRs reported potential sources of conflict of interest [21, 23]. A summary of the AMSTAR 2 results is shown in supplementary file 2. Overall, the methodological quality of the included SRs was unsatisfactory. According to statistical analysis, the consistency coefficient kappa between the two reviewers was 0.908 (*P* < 0.0001), which indicated that the evaluation of the two reviewers was independently balanced.

#### 3.2.2. Reporting Quality of Included SRs

**Figure 2:**
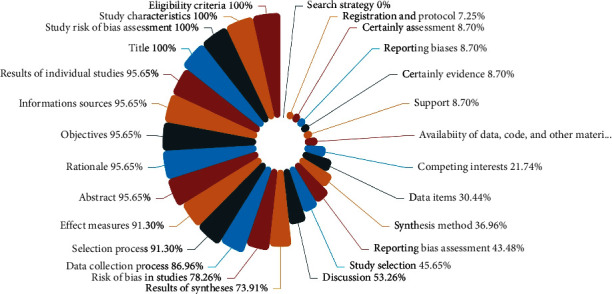
The proportion of the individual components of PRISMA of included SRs.

Table 3 shows the individual quality components of PRISMA. The consistency coefficient kappa between the two reviewers was 0.750 (*P* < 0.0001). Among the 23 SRs, the title, eligibility criteria, study characteristics, and introduction were all well-reported (100%). For the introduction of these SRs, 22 (95.65%) SRs [19–38, 40, 41] described the rationale for the review in the context of what is already known and provided precise and explicitly framing questions in objectives. For the methods of these SRs, the search strategy, data items, synthesis method, reporting bias assessment, and certainly assessment were reported inadequately. Regarding the search strategy, none of the SRs reported using filters or restrictions. Regarding the data items, more than half (60.87%) of the SRs [20–23, 29–32, 34, 37–41] listed and defined all outcome indicators, but none of the SRs listed and defined all other variables. Over 56% of the SRs [24–26, 29, 31–35, 37–39, 41]failed to describe the methods used to assess the risk of bias due to missing results in a synthesis, and over 90% of the SRs [19, 21, 23–41] failed to describe methods for evaluating the quality of evidence for each outcome. Regarding the results of the SRs, the flow diagram and text did not clearly describe the process of report selection in 2 (8.70%) SRs [24, 32]. Only 1 (4.35%) SR [20] provided the results of individual studies. Similarly, only 2 (8.70%) SRs presented reporting biases [20, 32] and 2 (8.70%) SRs showed certainly evidence [20, 22]. No sensitivity analysis was presented. For the discussion of these SRs, nearly all (95.65%) SRs [19–36, 38–41] discussed the limitations of the evidence and the implications of the findings for practice, policy, and future research. However, fewer than 15% of them interpreted the results based on other evidence and discussed limitations in the study process. In addition, 3 (13.04%) SRs [20, 22, 23] provided registration information. Only 1 (4.35%) SR [22] provided access to the protocol or stated that there was no protocol and described the relevant content and modification. Over 90% of these SRs [19, 20, 22, 24–41] failed to describe sources of funding and other support. The proportion of the individual components of PRISMA of included SRs is summarized in Figure 2. Overall, the reporting quality of the included SRs was low.

#### 3.2.3. Risk of Bias of Included SRs

**Figure 3:**
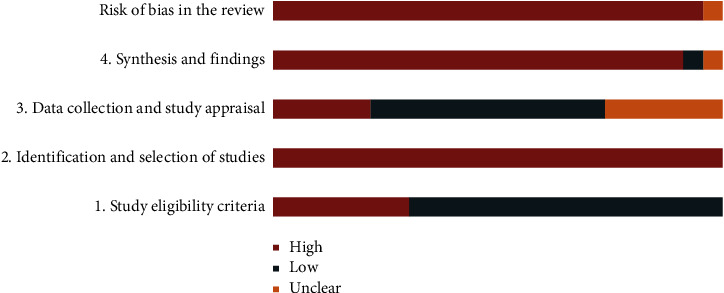
The result of the risk of bias of included SRs.

Figure 3 presents the results of the risk of bias of the included SRs by using the ROBIS tool, which contains 2 phases with 4 domains. Phase 1 was not performed in our study. Domain 1 assessed concerns regarding the specification of study eligibility criteria, and 16 of 23 (69.57%) SRs were rated as having a low risk of bias [19–21, 24–27, 29, 30, 32, 36–41]. Seven of 23 (30.43%) SRs were rated as having a high risk of bias [22, 23, 28, 31, 33–35]. Domain 2 assessed concerns regarding methods used to identify and select studies. All SRs (100%) were at high risk of bias. Domain 3 assessed concerns regarding methods used to collect data and appraise studies; 12 (52.17%) SRs had a low risk of bias [19–23, 25, 26, 29, 31, 36, 38, 41], 5 (21.74%) SRs had a high risk of bias [24, 32–35], and 6 (26.09%) SRs had an unclear risk of bias [27, 28, 30, 37, 39, 40]. Domain 4 assessed concerns regarding the synthesis and findings: 22 (95.65%) SRs had a high risk of bias [19, 20, 22–41], and 1 (4.35%) SR had an unclear risk of bias [21]. The individual quality components of ROBIS of included SRs are summarized in Table 4. The final phase considered the overall risk of bias of these SRs to be high. The consistency coefficient kappa between the two reviewers was 0.827 (*P* < 0.0001).

#### 3.2.4. Evidence Quality of Included SRs

**Figure 4:**
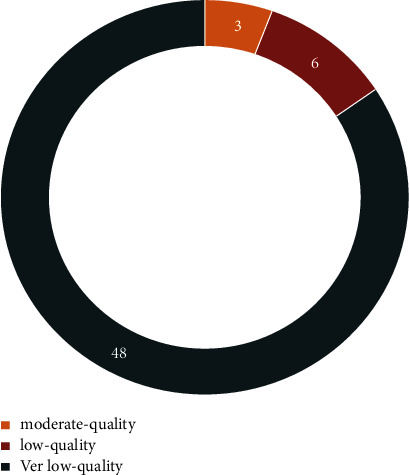
The evidence quality of included SRs.

This study includes 23 SRs involving 55 outcomes.  Table 5 shows the individual quality components of GRADE of included SRs. The consistency coefficient kappa between the two reviewers was 0.844 (*P* < 0.0001). For all outcomes, there were 3 outcomes with moderate-quality evidence [34], which included the total efficiency rate using the symptom score reducing index (SSRI), recurrence rate, and curing rate. The results of three moderate-quality studies showed that the total effective rate and curing rate of the acupuncture group were higher than those of the western medicine group, and the recurrence rate was lower than that of the western medicine group. There were 6 outcomes with low-quality evidence and 46 outcomes with very low-quality evidence.  Figure 4 presents the quality of the evidence for the included SRs. In addition, 14 outcomes had a large amount of heterogeneity (I^2^ > 50%), and 2 had a very large amount of heterogeneity (I^2^ > 80%). The elevated risk of bias, imprecision, and public bias were the main reasons for downgrading. Significant heterogeneity downgraded inconsistency, and imprecision was downgraded because the total sample size did not meet the optimal information size.

#### 3.2.5. Evidence from Quantitative Research Syntheses

In the 23 SRs, there were 11 outcome indicators, including total efficiency, adverse reaction rate, recurrence rate, clinical efficacy rate, improvement rate, curing rate, IgE levels, DLQI, QoL, total improvement in clinical signs and symptoms, and disease activity control. The types of intervention are acupuncture and related therapies, including bloodletting, cupping, auriculotherapy, acupoint injection, autohemotherapy, and catgut embedding. In the 23 SRs, the efficacy of acupuncture therapy in CU treatment (acupuncture alone or acupuncture-based combination therapies) yielded superior results to the control treatments. Considering the wide range of acupuncture and related therapies, we conducted a categorical analysis. When acupuncture was compared with antihistamines, one SR found no significant difference in the total effective rate (OR = 2.19, 95%CI (0.79 to 6.07), *P*=0.13, I^2^ = 47%) [40], and another SR reported the opposite conclusion (RR = 1.21, 95%CI (1.00 to 1.46), *P*=0.05) [19]. Additionally, the other two SRs found acupuncture to be more effective on global symptom improvement (RR = 1.37, 95%CI (1.11 to 1.70), *P*=0.003, I^2^ = 23%) [22] and curing rate (RR = 2.14, 95%CI (1.64 to 2.79), *P* < 0.0001, I^2^ = 32%) [24]. These 4 SRs also analyzed the efficacy between acupuncture with antihistamines and antihistamines, and the results showed that added acupuncture was more effective (RR = 1.77, 95%CI (1.41 to 2.22), *P* < 0.01, I^2^ = 0%; RR = 1.20, 95%CI (1.07 to 1.35), *P*=0.002, I^2^ = 0%; OR = 6.59, 95%CI (2.69 to 16.16), *P* < 0.0001, I^2^ = 0%; RR = 2.03, 95%CI (1.35 to 3.06), *P*=0.0006, I^2^ = 48%) [19, 22, 24, 40]. In addition, there was no statistical significance when cupping was compared with antihistamines (RR = 1.10, 95%CI (0.97 to 1.25), *P*=0.14, I^2^ = 52%) [23]. However, the efficacy of cupping combined with antihistamines was better than that of antihistamines alone (RR = 1.18, 95%CI (1.01 to 1.39), *P*=0.03, I^2^ = 67%) [23]. Autohemotherapy was the most frequently used therapy among the included SRs. Overall, 73.08% of these results were effective. Interestingly, when catgut embedding was compared with other therapies, the difference in efficacy was statistically significant (RR = 1.1, 95%CI (1.03 to 1.16), *P*=0.002, I^2^ = 30%) [26], but when it was combined with antihistamines, the result was reversed (RR = 1.14, 95%CI (0.98 to 1.34, *P*=0.10) [26]. This result may be related to the low sample size and low quality of the reviews. In addition, we also examined the efficacy of bloodletting, auriculotherapy, and acupoint injection. More details are provided in Table 6.

## 4. Discussion

When there is a need to quickly gather evidence to inform new policies or procedures, existing research syntheses are available [50–53]. SRs, which have the highest level of evidence, are increasingly used for evidence-based decision-making [53]. Currently, acupuncture in CU has been widely used in clinical practice in China [54]. A variety of SRs about CU interventions has been published with varying recommendations of treatment effectiveness. In this evidence-based review, we analyzed the evidence provided at the SR level of acupuncture and related therapies in CU and assessed the methodological and reporting quality of these SRs. The results showed that there were some deficiencies in the quality of methodology and reporting. The results of the AMSTAR 2 indicated that the authors should complete the protocol or register in the Cochrane Library or PROSPERO website in advance to reduce the risk of bias. Second, all SRs did not provide a list of excluded studies and justify the exclusions, which may lead to bias in the findings. In addition, the selection of study types for inclusion, the source of research funding for randomized trials in included SRs, and the potential impact of RoB in individual studies on the results of the meta-analysis were not well explained, which can directly reduce the rating overall confidence. Therefore, authors should adequately report these items in future studies. Notably, AMSTAR 2 was published in 2017. Considering that the majority of the included SRs in this study were published before 2017, the low quality of methodological evaluation may be related to a failure to meet the latest quality standards.

From the summary of PRISMA and ROBIS, some reporting shortcomings should be more noticeable in future research. Regarding the methods of the SRs, the authors should report the existence of the review protocol and full electronic search strategy for at least one major database, and they should describe the methods of analysis in detail. In addition, when reporting the results, the authors should assess ROB across studies and give results of additional analyses, if done (e.g., sensitivity or subgroup analyses, meta-regression). For the discussion of the SRs, it is suggested that the authors should assess the strength of evidence for each main outcome and consider their relevance to key groups. In addition, readers and decision-makers in health care can judge whether evaluation bias and conflicts of interest exist, and it is important for review authors to record the sources of funding reported in every study.

We summarized the current evidence of acupuncture and related therapies in CU for a wide range of outcome indicators. On the whole, compared with the control group, most of these results indicated that the experimental group was effective. According to the GRADE analysis, compared with the quality of different acupuncture treatments, the results of three moderate-quality outcomes showed that the total effective rate and curing rate of the acupuncture group were higher than those of the western medicine group [34], and the recurrence rate was lower than that of the western medicine group [34]. However, the evidence for other acupuncture treatments was low [24, 26, 27, 29, 31, 41] or very low-quality [19–33, 35–41], and no significant qualitative differences were found between the different treatment options. The factors that led to the (very) low-quality evidence may be as follows: the original studies were of poor quality, and all outcome measures were significantly at risk of bias due to the randomization, allocation concealment, and blinding of the included original studies. However, this has a certain relationship with the difficulty of acupuncture itself to strictly blind the patients. In addition, the research methods of these SRs were incomplete implementations. For 88% of the outcome indicators, the total sample size of the included SRs did not meet the optimal information size, and the confidence interval was wide, which seriously affected the imprecision of the outcome indicators. In addition, 82% of the outcome indicators have a large possibility of publication bias due to the small number of included primary studies and positive results.

The pathogenesis of CU is still unclear. At present, antihistamines are used to complete symptom control. However, many patients have an inadequate response to the majority of these drugs [14], which causes recurring attacks of CU. The above results can provide an evidence-based reference for acupuncture and related therapies in the treatment of CU. In clinical practice, when the patient is in an inadequate response to antihistamines, the doctors can choose to apply acupuncture and related therapies to treat CU based on comprehensive consideration of the patients' symptoms and preferences. When the symptoms of urticaria become recurrent or more severe, the evidence support that acupuncture and related therapies combined with another active therapy can improve symptom and reduce the recurrence rate [19–25, 27, 29, 34, 39, 40]. Additionally, the results suggest that we need to pay more attention to the quality of the primary study and strengthen training to meet relevant requirements on methodologies and reporting of the SRs and address these existing deficiencies of the SRs in future research.

Our study had some limitations. First, while all attempts were made to search and access all relevant literature, it is possible that some publications may have been missed in the search process due to language restrictions. Second, although uniform training was performed before our study, consistency analysis was performed after our study. The personal beliefs of authors can influence their judgment, and the study was also limited due to the subjectivity of quality evaluation.

## 5. Conclusions

Acupuncture and related therapies for the treatment of CU are supported by low-quality evidence-based medicine. However, considering the poor quality of these SRs, we suggest that studies with more rigorous designs, larger sample sizes, and higher methodological and reporting quality are necessary to provide stronger evidence.

## Figures and Tables

**Table 1:** Characteristics of included SRs.

Included study	Country	Age	Included study design	No. of study	No. of patient	Intervention	Comparator	Outcomes	Assessment of methodological quality	Meta-analysis conducted?	Subgroup	Sensitivity	Safety
Li [24] (2009)	China	Age ≥ 12 years old	RCT	12	984	Acupuncture therapy or combined with conventional medicine	Conventional medicine	(2) (3) (6)	Classified by the Cochrane handbook. The version is not stated and the Jadad scale evaluation	Yes	Yes	No	Yes

Yan [19] (2015)	China	Any age	RCT	8	755	Any acupoint stimulation or combined with other therapy	No treatment/placebo or western medication; same other therapy	(1) (8)	Classified by the Cochrane handbook. The version is not stated	Yes	Yes	No	Yes

Yao [22] (2016)	China	Any age	RCT	6	406	Acupuncture or combined with another active therapy	No treatment/placebo/sham acupuncture/other same active therapies	(1) (2) (3) (9)	Classified by the Cochrane handbook. The version is not stated	Yes	No	No	Yes

Liang [41] (2016)	China	Any age	RCT	15	1554	Autohemotherapy or combined with an antihistamine or other treatment	Antihistamine or acupuncture	(1) (2) (3) (7)	Classified by the Cochrane handbook. The version is not stated	Yes	Yes	No	Yes

Chen [33] (2016)	China	Any age	RCT	5	545	Autohemotherapy	Other therapies except for Autohemotherapy	(4)	Classified by the Cochrane handbook. The version is not stated	Yes	No	No	No

An [38] (2016)	China	Any age	RCT	12	988	Autohemotherapy	Other therapies except for autohemotherapy	(1) (3)	Jadad scale evaluation	Yes	Yes	Yes	No

Luo [39] (2016)	China	Any age	RCT	8	937	Autohemotherapy or combined with other therapies	Other therapies	(1) (3) (4)	Cochrane 5.0.1 and Jadad scale evaluation	Yes	Yes	Yes	Yes

Liu [35] (2017)	China	Any age	RCT/CCT	6	515	Autohemotherapy	Conventional western therapy	(4)	Jadad scale evaluation	Yes	No	No	No

Pu [28] (2017)	China	Any age	RCT	10	686	Autohemotherapy or combined with antihistamines	Antihistamine or acupuncture therapy	(2) (3) (4)	Cochrane 5.0.1	Yes	No	No	Yes

Zhang [31] (2018)	China	Any age	RCT	9	751	Acupoint embedding or combined with other therapies	Other therapies except for acupoint embedding	(1) (5) (6)	Classified by the Cochrane handbook. The version is not stated	Yes	No	Yes	Yes

Liang [32] (2018)	China	Any age	RCT/quasi-RCT/CCT	7	528	Acupoint embedding or combined with other therapies	Other therapies except for acupoint embedding	(4) (6)	Jadad scale evaluation	Yes	No	Yes	No

Zhu [21] (2018)	China	Any age	RCT	7	624	Auriculotherapy or combined with other therapies	Western medicine	(2) (4) (10)	Cochrane 5.0.1	Yes	No	Yes	Yes

Li [37] (2018)	China	Any age	RCT	14	1051	Autohemotherapy or combined with other therapies	Other therapies except for autohemotherapy	(1) (3) (6)	Classified by the Cochrane handbook. The version is not stated	Yes	No	Yes	Yes

Wei [26] (2019)	China	Any age	RCT	12	836	Acupoint embedding	Other therapies except for acupoint embedding	(2) (3) (4) (6)	Classified by the Cochrane handbook. The version is not stated	Yes	No	Yes	Yes

Zhao [40] (2019)	China	Age ≥ 18 years old	RCT	16	1131	Acupuncture/electroacupuncture (can be moxibustion, cupping, bleeding, etc.) or combined with the same intervention measures as the control group	Western medicine	(1) (3) (6) (7) (10)	Classified by the Cochrane handbook. The version is not stated	Yes	Yes	Yes	Yes

Li [36] (2019)	China	Any age	RCT	11	763	Autohemotherapy combined with other therapies	Other therapies except for autohemotherapy	(3) (6)	Classified by the Cochrane handbook. The version is not stated	Yes	No	No	Yes

Wu [29] (2019)	China	Any age	RCT	13	1608	Autohemotherapy combined with H1 receptor antagonists	H1 receptor antagonists	(1) (2) (3)	Cochrane 5.0.1	Yes	Yes	Yes	Yes

Zhao [30] (2019)	China	Any age	RCT/CCT	12	944	Autohemotherapy combined with acupuncture and other therapies	Other therapies except for autohemotherapy	(1) (2) (3) (4)	Cochrane 5.1.0 and Jadad scale evaluation	Yes	Yes	Yes	Yes

Yao [20] (2019)	China	Any age	RCT	7	512	Bloodletting therapy or combined with other active therapies	No treatment/placebo/sham bloodletting therapy/other same active therapies	(1) (2) (11)	Classified by the Cochrane handbook. The version is not stated	Yes	No	Yes	Yes

Zhao [27] (2020)	China	Age ≥ 18 years old	RCT	16	1131	Acupuncture or combined with western medicine	Western medicine	(1) (3) (10)	Classified by the Cochrane handbook. The version is not stated	Yes	No	Yes	No

Zhang [34] (2020)	China	Any age	RCT/quasi-RCT	16	1325	Acupuncture	Western medicine	(1) (3) (6)	Jadad scale evaluation	Yes	No	No	No

Xiao [23] (2020)	China	Any age	RCT	12	842	Dry/wet cupping or combination with other therapies	Other therapies	(1) (2) (3) (8)	Cochrane 5.0.1	Yes	Yes	No	Yes

Ke [25] (2021)	China	Any age	RCT	7	510	Acupuncture therapy combined with herbal decoction	Antihistamine	(1) (3) (4)	Classified by the Cochrane handbook. The version is not stated	Yes	No	Yes	Yes

*Note*. Outcomes: (1) total efficiency: according to the severity of clinical symptoms using 4 scores; the total score is the sum of the individual scores. Symptom Score Reducing Index (SSRI)=(total score before treatment-total score after treatment)/total score before treatment×100%; (2) adverse reaction rate; (3) recurrence rate; (4) clinical efficacy rate: the clinical complete recovery is considered to be effective, others are invalid; (5) improvement rate; (6) curing rate; (7) IgE levels; (8) the Dermatology Quality Life Index (DLQI); (9) quality of life score (QoL); (10) total improvement of clinical signs and symptoms; (11) disease activity control.

**Table 2:** The individual quality components of AMSTAR 2 of included SRs.

Included study	Item 1	Item 2^∗^	Item 3	Item 4^∗^	Item 5	Item 6	Item 7^∗^	Item 8	Item 9^∗^	Item 10	Item 11^∗^	Item 12	Item 13^∗^	Item 14	Item 15^∗^	Item 16	Ranking of quality
Li [24] (2009)	Y	N	N	Y	Y	Y	N	PY	PY	N	Y	N	N	N	N	N	⊕⊕⊕⊕
Yan [19] (2015)	Y	N	N	Y	N	Y	N	Y	PY	N	Y	Y	Y	Y	N	N	⊕⊕⊕⊕
Yao [22] (2016)	Y	Y	N	Y	Y	Y	N	PY	PY	N	Y	Y	Y	N	N	N	⊕⊕⊕⊕
Liang [41] (2016)	Y	N	N	PY	N	N	N	Y	PY	N	Y	Y	N	N	N	N	⊕⊕⊕⊕
Chen [33] (2016)	N	N	N	PY	Y	Y	N	PY	PY	N	Y	N	N	N	N	N	⊕⊕⊕⊕
An [38] (2016)	Y	N	N	Y	Y	Y	N	PY	PY	N	Y	Y	Y	N	N	N	⊕⊕⊕⊕
Luo [39] (2016)	Y	N	N	PY	N	N	N	Y	PY	N	Y	Y	Y	Y	Y	N	⊕⊕⊕⊕
Liu [35] (2017)	N	N	N	PY	N	N	N	N	PY	N	Y	N	N	Y	N	N	⊕⊕⊕⊕
Pu [28] (2017)	Y	N	N	Y	Y	Y	N	Y	PY	N	Y	Y	Y	N	Y	N	⊕⊕⊕⊕
Zhang [31] (2018)	Y	N	N	PY	Y	Y	N	PY	PY	N	Y	Y	N	N	N	N	⊕⊕⊕⊕
Liang [32] (2018)	Y	N	N	Y	Y	Y	N	Y	PY	N	Y	Y	N	N	N	N	⊕⊕⊕⊕
Zhu [21] (2018)	Y	N	N	Y	N	Y	N	Y	PY	N	Y	Y	N	N	N	Y	⊕⊕⊕⊕
Li [37] (2018)	Y	N	N	Y	Y	Y	N	Y	Y	N	Y	Y	N	N	N	N	⊕⊕⊕⊕
Wei [26] (2019)	Y	N	N	Y	Y	Y	N	Y	PY	N	Y	Y	Y	N	N	N	⊕⊕⊕⊕
Zhao [40] (2019)	Y	N	N	PY	Y	Y	N	Y	PY	N	Y	Y	Y	N	Y	N	⊕⊕⊕⊕
Li [36] (2019)	Y	N	N	PY	Y	Y	N	PY	PY	N	Y	Y	Y	N	Y	N	⊕⊕⊕⊕
Wu [29] (2019)	Y	N	N	PY	Y	Y	N	Y	Y	N	Y	Y	Y	N	Y	N	⊕⊕⊕⊕
Zhao [30] (2019)	Y	N	N	Y	Y	Y	N	Y	Y	N	Y	Y	Y	Y	Y	N	⊕⊕⊕⊕
Yao [20] (2019)	Y	N	N	Y	Y	Y	N	Y	PY	N	Y	Y	Y	N	N	N	⊕⊕⊕⊕
Zhao [27] (2020)	Y	N	N	PY	Y	Y	N	Y	PY	N	Y	Y	N	N	N	N	⊕⊕⊕⊕
Zhang [34] (2020)	Y	N	N	PY	N	N	N	Y	PY	N	Y	Y	N	N	N	N	⊕⊕⊕⊕
Xiao [23] (2020)	N	PY	N	PY	Y	Y	N	Y	PY	N	Y	Y	Y	Y	N	Y	⊕⊕⊕⊕
Ke [25] (2021)	Y	N	N	PY	Y	Y	N	PY	PY	N	Y	Y	N	N	N	N	⊕⊕⊕⊕

Note: ^∗^; The key items of the AMSTAR 2. ⊕ represents the ranking of quality as high, ⊕⊕ represents the ranking of quality as moderate, ⊕⊕⊕ represents the ranking of quality as low, and ⊕⊕⊕⊕ represents the ranking of quality as critically low. Item1: did the research questions and inclusion criteria for the review include the components of PICO? Item2: did the report of the review contain an explicit statement that the review methods were established prior to the conduct of the review and did the report justify any significant deviations from the protocol? Item3: did the review authors explain their selection of the study designs for inclusion in the review? Item4: did the review authors use a comprehensive literature search strategy? Item5: did the review authors perform study selection in duplicate? Item6: did the review authors perform data extraction in duplicate? Item7: did the review authors provide a list of excluded studies and justify the exclusions? Item8: did the review authors describe the included studies in adequate detail? Item9: did the review authors use a satisfactory technique for assessing the risk of bias (RoB) in individual studies that were included in the review? Item10: did the review authors report on the sources of funding for the studies included in the review? Item11: if a meta-analysis was performed, did the review authors use appropriate methods for the statistical combination of results? Item12: if a meta-analysis was performed, did the review authors assess the potential impact of RoB in individual studies on the results of the meta-analysis or another evidence synthesis? Item13: did the review authors account for RoB in primary studies when interpreting/discussing the results of the review? Item14: did the review authors provide a satisfactory explanation for, and discussion of, any heterogeneity observed in the results of the review? Item15: if they performed quantitative synthesis did the review authors carry out an adequate investigation of publication bias (small study bias) and discuss its likely impact on the results of the review? Item16: did the review authors report any potential sources of conflict of interest, including any funding they received for conducting the review? Y: yes; N: no; PY: partial yes.

**Table 3:** The individual quality components of PRISMA of included SRs.

Items	Selection and topic		Li [24] (2009)	Yan [19] (2015)	Yao [22] (2016)	Liang [41] (2016)	Chen [33] (2016)	An [38] (2016)	Luo [39] (2016)	Liu [35] (2017)	Pu [28] (2017)	Zhang [31] (2018)	Liang [32] (2018)	Zhu [21] (2018)
1	Title	Title	Y	Y	Y	Y	Y	Y	Y	Y	Y	Y	Y	Y
2	Abstract	Abstract	Y	Y	Y	Y	Y	Y	Y	Y	Y	Y	Y	N
3	Introduction	Rationale	Y	Y	Y	Y	Y	Y	N	Y	Y	Y	Y	Y
4		Objectives	Y	Y	Y	Y	Y	Y	Y	N	Y	Y	Y	Y
5	Methods	Eligibility criteria	Y	Y	Y	Y	Y	Y	Y	Y	Y	Y	Y	Y
6		Information sources	Y	Y	Y	Y	Y	Y	Y	Y	Y	Y	Y	N
7		Search strategy	N	N	N	N	N	N	N	N	N	N	N	N
8		Selection process	Y	Y	Y	Y	Y	Y	N	Y	Y	Y	Y	N
9		Data collection process	Y	Y	Y	Y	N	Y	N	N	Y	Y	Y	Y
10a		Data items	N	N	Y	Y	N	Y	Y	N	N	Y	Y	Y
10b			N	N	N	N	N	N	N	N	N	N	N	N
11		Study risk of bias assessment	Y	Y	Y	Y	Y	Y	Y	Y	Y	Y	Y	Y
12		Effect measures	Y	Y	Y	Y	Y	Y	N	Y	Y	N	Y	Y
13a		Synthesis method	N	N	N	N	N	N	N	N	N	N	N	Y
13b			N	N	N	N	N	N	N	N	N	N	N	N
13c			N	N	N	N	N	N	N	N	N	N	N	N
13d			N	Y	Y	Y	Y	Y	N	Y	Y	Y	Y	Y
13e			N	Y	Y	Y	Y	Y	N	Y	Y	Y	Y	Y
13f			N	N	Y	N	N	N	Y	N	N	N	N	Y
14		Reporting bias assessment	N	Y	Y	N	N	N	N	N	Y	N	N	Y
15		Certainly assessment	N	N	Y	N	N	N	N	N	N	N	N	N
16a	Result	Study selection	N	Y	Y	Y	Y	Y	Y	Y	Y	Y	N	Y
16b			N	N	N	N	N	N	N	N	N	N	N	N
17		Study characteristics	Y	Y	Y	Y	Y	Y	Y	Y	Y	Y	Y	Y
18		Risk of bias in studies	N	Y	Y	Y	N	Y	Y	N	Y	Y	N	Y
19		Results of individual studies	Y	Y	Y	Y	Y	Y	Y	Y	Y	Y	Y	Y
20a		Results of syntheses	Y	Y	Y	Y	Y	Y	Y	Y	Y	Y	N	Y
20b			Y	Y	Y	Y	Y	Y	Y	Y	Y	Y	Y	Y
20c			Y	Y	Y	Y	N	Y	Y	N	Y	N	N	Y
20d			N	N	N	N	N	Y	Y	N	Y	N	N	N
21		Reporting biases	N	N	N	N	N	N	N	N	N	N	Y	N
22		Certainly evidence	N	N	Y	N	N	N	N	N	N	N	N	N
23a	Discussion	Discussion	N	Y	N	N	N	N	N	N	N	N	N	N
23b			Y	Y	Y	Y	Y	Y	Y	Y	Y	Y	Y	Y
23c			N	N	N	N	N	N	N	N	N	N	N	N
23d			Y	Y	Y	Y	Y	Y	Y	Y	Y	Y	Y	Y
24a	Other information	Registration and protocol	N	N	Y	N	N	N	N	N	N	N	N	N
24b			N	N	Y	N	N	N	N	N	N	N	N	N
24c			N	N	Y	N	N	N	N	N	N	N	N	N
25		Support	N	N	N	N	N	N	N	N	N	N	N	Y
26		Competing interests	N	Y	Y	N	N	N	N	N	N	N	N	Y
27		Availability of data, code, and other materials	N	N	Y	N	N	N	N	N	N	N	N	N

Items	Selection and topic		Li [37] (2018)	Wei [26] (2019)	Zhao [40] (2019)	Li [36] (2019)	Wu [29] (2019)	Zhao [30] (2019)	Yao [20] (2019)	Zhao [27] (2020)	Zhang [34] (2020)	Xiao [23] (2020)	Ke [25] (2021)	Compliance (%)
1	Title	Title	Y	Y	Y	Y	Y	Y	Y	Y	Y	Y	Y	100%
2	Abstract	Abstract	Y	Y	Y	Y	Y	Y	Y	Y	Y	Y	Y	95.65%
3	Introduction	Rationale	Y	Y	Y	Y	Y	Y	Y	Y	Y	Y	Y	95.65%
4		Objectives	Y	Y	Y	Y	Y	Y	Y	Y	Y	Y	Y	95.65%
5	Methods	Eligibility criteria	Y	Y	Y	Y	Y	Y	Y	Y	Y	Y	Y	100%
6		Information sources	Y	Y	Y	Y	Y	Y	Y	Y	Y	Y	Y	95.65%
7		Search strategy	N	N	N	N	N	N	N	N	N	N	N	0%
8		Selection process	Y	Y	Y	Y	Y	Y	Y	Y	Y	Y	Y	91.30%
9		Data collection process	Y	Y	Y	Y	Y	Y	Y	Y	Y	Y	Y	86.96%
10a		Data items	Y	N	Y	N	Y	Y	Y	N	Y	Y	N	60.87%
10b			N	N	N	N	N	N	N	N	N	N	N	0%
11		Study risk of bias assessment	Y	Y	Y	Y	Y	Y	Y	Y	Y	Y	Y	100%
12		Effect measures	Y	Y	Y	Y	Y	Y	Y	Y	Y	Y	Y	91.30%
13a		Synthesis method	N	N	Y	N	N	N	N	N	N	N	N	8.70%
13b			N	N	N	N	N	N	Y	N	N	N	N	4.35%
13c			N	N	Y	N	N	N	N	N	N	N	N	4.35%
13d			Y	Y	Y	Y	Y	Y	Y	Y	Y	Y	Y	91.30%
13e			Y	Y	Y	Y	Y	Y	Y	N	Y	Y	Y	86.96%
13f			N	Y	Y	N	Y	N	N	N	N	N	N	26.08%
14		Reporting bias assessment	N	N	Y	Y	N	Y	Y	Y	N	Y	N	43.48%
15		Certainly assessment	N	N	N	N	N	N	Y	N	N	N	N	8.70%
16a	Result	Study selection	Y	Y	Y	Y	Y	Y	Y	Y	Y	Y	Y	91.30%
16b			N	N	N	N	N	N	N	N	N	N	N	0%
17		Study characteristics	Y	Y	Y	Y	Y	Y	Y	Y	Y	Y	Y	100%
18		Risk of bias in studies	Y	Y	Y	Y	Y	Y	Y	Y	N	Y	Y	78.26%
19		Results of individual studies	Y	Y	Y	Y	Y	Y	N	Y	Y	Y	Y	95.65%
20a		Results of syntheses	Y	Y	Y	Y	Y	Y	Y	Y	Y	Y	Y	95.65%
20b			Y	Y	Y	Y	Y	Y	Y	Y	Y	Y	Y	100%
20c			N	Y	Y	N	Y	Y	N	Y	N	Y	N	60.87%
20d			Y	Y	Y	N	Y	Y	N	Y	N	N	N	39.13%
21		Reporting biases	N	N	N	N	N	N	Y	N	N	N	N	8.70%
22		Certainly evidence	N	N	N	N	N	N	Y	N	N	N	N	8.70%
23a	Discussion	Discussion	N	N	Y	N	N	N	N	N	N	Y	N	13.04%
23b			N	Y	Y	Y	Y	Y	Y	Y	Y	Y	Y	95.65%
23c			N	Y	N	N	N	N	Y	N	N	N	N	8.70%
23d			Y	Y	N	Y	Y	Y	Y	Y	Y	Y	Y	95.65%
24a	Other information	Registration and protocol	N	N	N	N	N	N	Y	N	N	Y	N	13.04%
24b			N	N	N	N	N	N	N	N	N	N	N	4.35%
24c			N	N	N	N	N	N	N	N	N	N	N	4.35%
25		Support	N	N	N	N	N	N	N	N	N	Y	N	8.70%
26		Competing interests	N	N	N	N	N	N	Y	N	N	Y	N	21.74%
27		Availability of data, code, and other materials	N	N	Y	N	N	N	Y	N	N	N	N	13.04%

**Table 4:** The individual quality components of ROBIS of included SRs.

Included study	Phase 2				Phase 3
	1. Study eligibility criteria	2. Identification and selection of studies	3. Data collection and study appraisal	4. Synthesis and findings	Risk of bias in the review
Li [24] (2009)	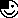	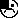	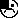	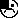	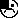
Yan [19] (2015)	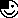	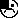	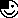	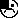	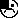
Yao [22] (2016)	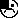	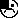	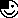	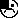	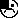
Liang [41] (2016)	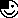	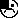	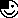	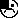	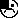
Chen [33] (2016)	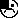	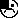	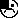	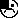	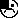
An [38] (2016)	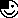	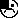	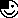	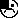	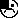
Luo [39] (2016)	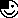	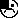	?	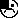	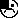
Liu [35] (2017)	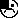	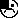	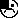	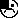	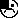
Pu [28] (2017)	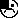	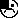	?	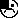	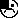
Zhang [31] (2018)	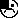	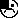	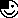	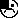	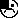
Liang [32] (2018)	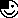	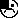	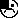	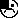	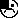
Zhu [21] (2018)	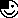	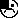	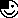	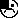	?
Li [37] (2018)	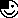	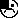	?	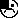	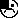
Wei [26] (2019)	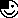	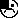	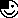	?	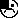
Zhao [40] (2019)	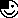	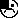	?	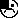	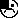
Li [36] (2019)	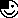	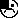	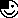	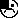	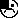
Wu [29] (2019)	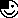	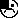	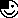	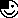	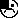
Zhao [30] (2019)	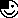	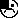	?	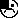	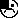
Yao [20] (2019)	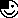	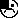	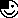	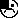	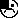
Zhao [27] (2020)	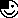	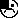	?	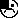	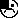
Zhang [34] (2020)	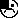	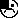	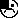	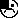	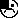
Xiao [23] (2020)	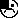	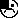	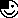	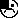	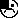
Ke [25] (2021)	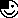	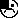	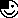	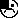	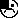

Note: 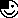 = low risk; 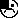 = high risk; ? = unclear risk.

**Table 5:** The individual quality components of GRADE of included SRs.

Included study	Type of Study	Outcomes	Downgrading factors					Outcome
			Risk of bias	Inconsistency	Indirectness	Imprecision	Publication bias	Quality of evidence
Li [24] (2009)	RCT	(6)^∗^	serious	no	no	serious	strongly suspected	⊕⊕⊕⊕
		(3)	serious	no	no	no	strongly suspected	⊕⊕⊕
Yan [19] (2015)	RCT	(1)^∗^	serious	no	no	serious	strongly suspected	⊕⊕⊕⊕
		(8)	serious	no	no	serious	strongly suspected	⊕⊕⊕⊕
Yao [22] (2016)	RCT	(1)^∗^	serious	no	no	serious	strongly suspected	⊕⊕⊕⊕
Liang [41] (2016)	RCT	(1)^∗^	serious	very serious	no	serious	strongly suspected	⊕⊕⊕⊕
		(2)	serious	no	no	no	strongly suspected	⊕⊕⊕
		(3)	serious	no	no	serious	strongly suspected	⊕⊕⊕⊕
		(7)	serious	very serious	no	serious	strongly suspected	⊕⊕⊕⊕
Chen [33] (2016)	RCT	(4)^∗^	serious	no	no	serious	strongly suspected	⊕⊕⊕⊕
An [38] (2016)	RCT	(1)^∗^	very serious	serious	no	serious	strongly suspected	⊕⊕⊕⊕
		(3)	very serious	serious	no	serious	strongly suspected	⊕⊕⊕⊕
Luo [39] (2016)	RCT	(1)^∗^	very serious	serious	no	serious	strongly suspected	⊕⊕⊕⊕
		(3)	very serious	serious	no	serious	strongly suspected	⊕⊕⊕⊕
		(4)	very serious	serious	no	serious	strongly suspected	⊕⊕⊕⊕
Liu [35] (2017)	RCT/CCT	(4)^∗^	serious	serious	no	serious	strongly suspected	⊕⊕⊕⊕
Pu [28] (2017)	RCT	(3)	serious	no	no	serious	strongly suspected	⊕⊕⊕⊕
		(4)	serious	no	no	serious	strongly suspected	⊕⊕⊕⊕
Zhang [31] (2018)	RCT	(1)^∗^	serious	no	no	serious	strongly suspected	⊕⊕⊕⊕
		(5)	serious	no	no	no	strongly suspected	⊕⊕⊕
		(6)	serious	no	no	serious	strongly suspected	⊕⊕⊕⊕
Liang [32] (2018)	RCT/quasi-RCT/CCT	(4)^∗^	serious	no	no	serious	strongly suspected	⊕⊕⊕⊕
		(6)	serious	no	no	serious	strongly suspected	⊕⊕⊕⊕
Zhu [21] (2018)	RCT	(4)	serious	no	no	serious	strongly suspected	⊕⊕⊕⊕
		(10)^∗^	serious	serious	no	serious	strongly suspected	⊕⊕⊕⊕
Li [37] (2018)	RCT	(1)^∗^	serious	serious	no	serious	strongly suspected	⊕⊕⊕⊕
		(3)	serious	no	no	serious	strongly suspected	⊕⊕⊕⊕
		(6)	serious	serious	no	serious	strongly suspected	⊕⊕⊕⊕
Wei [26] (2019)	RCT	(3)	serious	no	no	serious	strongly suspected	⊕⊕⊕⊕
		(4)^∗^	serious	no	no	serious	strongly suspected	⊕⊕⊕⊕
		(6)	serious	no	no	serious	undetected	⊕⊕⊕
Zhao [40] (2019)	RCT	(1)^∗^	serious	no	no	serious	strongly suspected	⊕⊕⊕⊕
		(3)	serious	no	no	serious	strongly suspected	⊕⊕⊕⊕
		(6)	serious	no	no	serious	strongly suspected	⊕⊕⊕⊕
		(7)	serious	no	no	serious	strongly suspected	⊕⊕⊕⊕
		(10)	serious	serious	no	serious	strongly suspected	⊕⊕⊕⊕
Li [36] (2019)	RCT	(3)	serious	no	no	serious	strongly suspected	⊕⊕⊕⊕
		(6)^∗^	serious	no	no	serious	strongly suspected	⊕⊕⊕⊕
Wu [29] (2019)	RCT	(1)^∗^	serious	serious	no	serious	undetected	⊕⊕⊕⊕
		(3)	serious	no	no	serious	undetected	⊕⊕⊕
Zhao [30] (2019)	RCT/CCT	(1)^*∗*^	serious	no	no	serious	strongly suspected	⊕⊕⊕⊕
		(3)	serious	no	no	serious	strongly suspected	⊕⊕⊕⊕
		(4)	serious	no	no	serious	strongly suspected	⊕⊕⊕⊕
Yao [20] (2019)	RCT	(1)	serious	serious	no	serious	strongly suspected	⊕⊕⊕⊕
		(11)^*∗*^	serious	no	no	serious	strongly suspected	⊕⊕⊕⊕
Zhao [27] (2020)	RCT	(1)^*∗*^	serious	no	no	serious	strongly suspected	⊕⊕⊕⊕
		(3)	serious	no	no	no	strongly suspected	⊕⊕⊕
		(10)	serious	serious	no	serious	strongly suspected	⊕⊕⊕⊕
Zhang [34] (2020)	RCT/quasi-RCT	(1)^*∗*^	serious	no	no	no	undetected	⊕⊕
		(3)	serious	no	no	no	undetected	⊕⊕
		(6)	serious	no	no	no	undetected	⊕⊕
Xiao [23] (2020)	RCT	(1)^*∗*^	very serious	serious	no	serious	undetected	⊕⊕⊕⊕
		(3)	very serious	no	no	serious	undetected	⊕⊕⊕⊕
Ke [25] (2021)	RCT	(1)^*∗*^	serious	no	no	serious	undetected	⊕⊕⊕⊕
		(4)	serious	no	no	serious	undetected	⊕⊕⊕⊕

Note: ^∗^ represents the primary outcome measure, which determines the overall quality of the article; ⊕ represents the ranking of quality as high, ⊕⊕ represents the ranking of quality as moderate, ⊕⊕⊕ represents the ranking of quality as low, and ⊕⊕⊕⊕ represents the ranking of quality as very low. (1) Risk of Bias: the included study has large biases in terms of randomization, allocation concealment, blinding, and loss of follow-up. (2) Inconsistency: the overlapping of confidence intervals of different studies is poor, and the I^2^ value of the combined results is large. (3) Indirectness: differences in populations, interventions, and outcomes. (4) Imprecision: the sample size of the included studies was too small and the confidence interval was wide. (5) Publication bias: funnel diagram shows asymmetry, gray literature was not retrieved and the search database is incomplete.Outcomes: (1) total efficiency: according to the severity of clinical symptoms using 4 scores; the total score is the sum of the individual scores. Symptom Score Reducing Index (SSRI) = (total score before treatment-total score after treatment)/total score before treatment×100%; (2) adverse reaction rate; (3) recurrence rate; (4) clinical efficacy rate: the clinical complete recovery is considered to be effective, others are invalid; (5) improvement rate; (6) curing rate; (7) IgE levels; (8) the Dermatology Quality Life Index (DLQI); (9) quality of life score (QoL); (10) total improvement of clinical signs and symptoms; (11) disease activity control.

**Table 6:** Summary of evidence of included SRs.

Included study	Outcomes	GRADE assessment					Relative effect (95% CI)	*P* value	Certainty
		Risk of bias	Inconsistency	Indirectness	Imprecision	Publication bias			
*Acupuncture or electroacupuncture VS Antihistamine*									

Li [24] (2009)	Curing rate	serious	No	no	serious	strongly suspected	RR = 2.14, 95%CI (1.64 to 2.79)	*P* < 0.0001	VERY LOW
Yao [22] (2016)	Total effective rate	serious	No	no	serious	strongly suspected	RR = 1.37, 95%CI (1.11 to 1.70)	*P*=0.003	VERY LOW
Yan [19] (2015)	Total effective rate	serious	No	no	serious	strongly suspected	RR = 1.21, 95%CI (1.00 to 1.46)	*P*=0.05	VERY LOW
Zhao [40] (2019)	Total effective rate	serious	No	no	serious	strongly suspected	OR = 2.19, 95%CI (0.79 to 6.07)	*P*=0.13	VERY LOW

*Acupuncture or electroacupuncture combined with antihistamine VS Antihistamine*									

Li [24] (2009)	Curing rate	serious	No	no	serious	strongly suspected	RR = 2.03, 95%CI (1.35 to 3.06)	*P*=0.0006	VERY LOW
	Recurrence rate	serious	No	no	no	strongly suspected	RR = 0.35, 95%CI (0.13 to 0.93)	*P*=0.03	LOW
Yao [22] (2016)	Total effective rate	serious	no	no	serious	strongly suspected	RR = 1.77, 95%CI (1.41 to 2.22)	*P* < 0.01	VERY LOW
Yan [19] (2015)	Total effective rate	serious	no	no	serious	strongly suspected	RR = 1.20, 95%CI (1.07 to 1.35)	*P*=0.002	VERY LOW
Zhao [40] (2019)	Total effective rate	serious	no	no	serious	strongly suspected	OR = 6.59, 95%CI (2.69 to 16.16)	*P* < 0.0001	VERY LOW

*Acupuncture combined with other therapies VS Antihistamine*									

Ke [25] (2021)	Total effective rate	serious	no	no	serious	undetected	RR = 1.35, 95%CI (1.24 to 1.47)	*P* < 0.0001	VERY LOW
Zhao [40] (2019)	Total effective rate	serious	no	no	serious	strongly suspected	OR = 4.35, 95%CI (2.41 to 7.86)	*P* < 0.0001	VERY LOW
	Curing rate	serious	no	no	serious	strongly suspected	OR = 2.44, 95%CI (1.80 to 3.31)	*P* < 0.0001	VERY LOW
	IgE	serious	no	no	serious	strongly suspected	SMD = -1.71, 95%CI (-2.12 to -1.29)	*P* < 0.0001	VERY LOW
	Recurrence rate	serious	no	no	serious	strongly suspected	OR = 0.28, 95%CI (0.14 to 0.55)	*P*=0.0003	VERY LOW
Zhao [27] (2020)	Total effective rate	serious	no	no	serious	strongly suspected	RR = 3.85, 95%CI (2.61 to 5.69)	*P* < 0.0001	VERY LOW
	Recurrence rate	serious	no	no	no	strongly suspected	OR = 0.28, 95%CI (0.14 to 0.55)	*P*=0.0003	LOW

*Acupuncture or Acupuncture combined with other therapies vs. Western medicine*									

Zhang [34] (2020)	Total effective rate	serious	no	no	no	undetected	OR = 3.86, 95%CI (2.71 to 5.49)	*P* < 0.0001	MODERATE
	Recurrence rate	serious	no	no	no	undetected	OR = 0.34, 95%CI (0.16 to 0.72)	*P*=0.005	MODERATE
	Curing rate	serious	no	no	no	undetected	OR = 2.23, 95%CI (1.75 to 2.85)	*P* < 0.0001	MODERATE

*Bloodletting VS Antihistamine*									

Yao [20] (2019)	Disease activity control	serious	no	no	serious	strongly suspected	MD = 0.67, 95%CI (0.03 to 1.31)	*P*=0.04	VERY LOW
	Total effective rate	serious	no	no	serious	strongly suspected	RR = 1.10, 95%CI (0.97 to 1.26)	*P*=0.15	VERY LOW

*Bloodletting combined with antihistamine VS Antihistamine*									

Yao [20] (2019)	Total effective rate	serious	no	no	serious	strongly suspected	RR = 1.34, 95%CI (1.10 to 1.63)	*P*=0.003	VERY LOW

*Cupping VS Antihistamine*									

Xiao [23] (2020)	Total effective rate	very serious	serious	no	serious	undetected	RR = 1.10, 95%CI (0.97 to 1.25)	*P*=0.14	VERY LOW
	Recurrence rate	very serious	no	no	serious	undetected	RR = 0.56, 95%CI (0.23 to 1.36)	*P*=0.20	VERY LOW

*Cupping combined with antihistamine VS Antihistamine*									

Xiao [23] (2020)	Total effective rate	very serious	serious	no	serious	undetected	RR = 1.18, 95%CI (1.01 to 1.39)	*P*=0.03	VERY LOW
	Recurrence rate	very serious	no	no	serious	undetected	RR = 0.52, 95%CI (0.32 to 0.84)	*P*=0.007	VERY LOW

*Cupping combined with acupuncture VS Acupuncture*									

Xiao [23] (2020)	Total effective rate	very serious	serious	no	serious	undetected	RR = 1.25, 95%CI (1.07 to 1.46)	*P*=0.006	VERY LOW

*Autohemotherapy vs. Placebo*									

Liang [41] (2016)	Total effective rate	serious	very serious	no	serious	strongly suspected	RR = 1.51, 95%CI (1.06 to 2.14)	*P*=0.02	VERY LOW

*Autohemotherapy vs. Antihistamine*									

Luo [39] (2016)	Total effective rate	very serious	serious	no	serious	strongly suspected	RR = 1.05, 95%CI (0.97 to 1.13)	*P*=0.21	VERY LOW
	Clinical efficacy rate	very serious	serious	no	serious	strongly suspected	RR = 1.12, 95%CI (0.98 to 1.28)	*P*=0.21	VERY LOW
Liang [41] (2016)	Total effective rate	serious	very serious	no	serious	strongly suspected	RR = 1.14, 95%CI (1.04 to 1.26)	*P*=0.05	VERY LOW
	IgE	serious	very serious	no	serious	strongly suspected	RR = -11.15, 95%CI (-55.62 to 33.32)	*P*=0.62	VERY LOW
Zhao [30] (2019)	Total effective rate	serious	no	no	serious	strongly suspected	RR = 1.28, 95%CI (1.17 to 1.40)	*P* < 0.00001	VERY LOW
	Curing rate	serious	no	no	serious	strongly suspected	RR = 1.27, 95%CI (1.13 to 1.44)	*P* < 0.0001	VERY LOW
	Recurrence rate	serious	no	no	serious	strongly suspected	RR = 0.34, 95%CI (0.26 to 0.46)	*P* < 0.00001	VERY LOW

*Autohemotherapy vs. Other treatment*									

An [38] (2016)	Total effective rate	very serious	serious	no	serious	strongly suspected	RR = 1.08, 95%CI (1.12 to 1.15)	*P*=0.006	VERY LOW
	Recurrence rate	very serious	serious	no	serious	strongly suspected	RR = 0.46, 95%CI (0.26 to 0.81)	*P*=0.007	VERY LOW
Luo [39] (2016)	Total effective rate	very serious	serious	no	serious	strongly suspected	RR = 1.07, 95%CI (0.88 to 1.29)	*P*=0.51	VERY LOW
	Clinical efficacy rate	very serious	serious	no	serious	strongly suspected	RR = 1.36, 95%CI (0.95 to 1.96)	*P*=0.10	VERY LOW
Pu [28] (2017)	Clinical efficacy rate	serious	no	no	serious	strongly suspected	RR = 1.13, 95%CI (1.07 to 1.19)	*P* < 0.00001	VERY LOW
	Recurrence rate	serious	no	no	serious	strongly suspected	RR = 0.32, 95%CI (0.21 to 0.48)	*P* < 0.00001	VERY LOW
Zhao [30] (2019)	Total effective rate	serious	no	no	serious	strongly suspected	RR = 1.28, 95%CI (1.11 to 1.47)	*P*=0.0006	VERY LOW
	Curing rate	serious	no	no	serious	strongly suspected	RR = 1.33, 95%CI (1.05 to 1.69)	*P*=0.02	VERY LOW

*Autohemotherapy combined with antihistamine vs. Antihistamine*									

Yan [19] (2015)	Total effective rate	serious	no	no	serious	strongly suspected	RR = 1.06, 95%CI (0.98 to 1.14)	*P*=0.13	VERY LOW
	Dermatology Quality Life Index	serious	no	no	serious	strongly suspected	MD = 0.90, 95%CI (1.34 to 0.46)	*P* < 0.0001	VERY LOW
Luo [39] (2016)	Recurrence rate	very serious	serious	no	serious	strongly suspected	RR = 0.10, 95%CI (0.98 to 1.28)	*P* < 0.00001	VERY LOW
Liang [41] (2016)	Total effective rate	serious	very serious	no	serious	strongly suspected	RR = 1.09, 95%CI (0.93 to 1.27)	*P*=0.27	VERY LOW
	Recurrence rate	serious	no	no	serious	strongly suspected	RR = 0.36, 95%CI (0.23 to 0.55)	*P* < 0.0001	VERY LOW
Wu [29] (2019)	Total effective rate	serious	serious	no	serious	undetected	RR = 1.25, 95%CI (1.19 to 1.32)	*P* < 0.001	VERY LOW
	Recurrence rate	serious	no	no	serious	undetected	RR = 0.30, 95%CI (0.22 to 0.39)	*P* < 0.001	LOW

*Autohemotherapy combined with herbal medicine vs. Herbal medicine*									

Yan [19] (2015)	Total effective rate	serious	no	no	serious	strongly suspected	RR = 1.30, 95% CI (1.10, 1.55)	*P*=0.002	VERY LOW
Luo [39] (2016)	Total effective rate	very serious	serious	no	serious	strongly suspected	RR = 1.30, 95% CI (1.10, 1.55)	*P*=0.002	VERY LOW
	Clinical efficacy rate	very serious	serious	no	serious	strongly suspected	RR = 1.52, 95% CI (1.20, 1.94)	*P*=0.0006	VERY LOW

*Catgut embedding vs. Other therapy*									

Wei [26] (2019)	Clinical efficacy rate	serious	no	no	serious	strongly suspected	RR = 1.1, 95% CI (1.03 to 1.16)	*P*=0.002	VERY LOW
	Curing rate	serious	no	no	serious	undetected	RR = 1.59, 95%CI (1.30 to 1.95)	*P* < 0.00001	LOW
	Recurrence rate	serious	no	no	serious	strongly suspected	RR = 0.49, 95%CI (0.27 to 0.86)	*P*=0.01	VERY LOW

*Catgut embedding combined with antihistamine vs. Antihistamine*									

Yan [19] (2015)	Total effective rate	serious	no	no	serious	strongly suspected	RR = 1.14, 95%CI (0.98 to 1.34)	*P*=0.10	VERY LOW

*Auriculotherapy or auriculotherapy combined with other therapies vs. Western medicine*									

Zhu [21] (2018)	Improving clinical signs and symptoms	serious	serious	no	serious	strongly suspected	OR = 0.74, 95%CI (0.35 to 1.56)	*P*=0.42	VERY LOW
	Clinical efficacy rate	serious	no	no	serious	strongly suspected	OR = 3.81, 95%CI (2.07 to 7.01)	*P* < 0.0001	VERY LOW

*Acupoint injection combined with western medicine vs. Western medicine*									

Yan [19] (2015)	Total effective rate	serious	no	no	serious	strongly suspected	RR = 1.07, 95%CI (0.99 to 1.17)	*P*=0.09	VERY LOW

*Note.* CI, confidence interval; OR, odds ratio; RR, relative risk; MD, mean difference; SMD, standardized mean difference.

## Data Availability

All relevant data are within the paper and its supporting information files.

## Supplementary Materials

Supplementary Materials Supplemental 1: search strategy for PubMed. Supplemental 2: the summary of the AMSTAR 2 results.()
