# Supplementary material for: Acupuncture and Related Therapies for Chronic Urticaria: A Critical Overview of Systematic Reviews
Source: Evid Based Complement Alternat Med. 2022 Oct 27;2022:2094589. doi: 10.1155/2022/2094589 (PMC9633187; doi:10.1155/2022/2094589)
Supplement: Supplementary Materials — Supplemental 1: search strategy for PubMed. Supplemental 2: the summary of the AMSTAR 2 results.() [file 2094589.f1.zip › Supplemental 1 Search strategy.docx]

**Supplemental 1: Search strategy**

| **Supplemental 1.1: Search strategy for PubMed** | |
| --- | --- |
| **Sequence** | **Search term** |
| #1 | “urticaria”[Mesh] |
| #2 | “chronic urticaria” |
| #3 | “hives” |
| #4 | “nettle-rash” |
| #5 | “Angioedema” |
| #6 | “Wind cluster” |
| #7 | #1 OR #2 OR #3 OR #4 OR #5 OR #6 |
| #8 | “systematic review”[Mesh] |
| #9 | “meta analysis” |
| #10 | #8 OR #9 |
| #11 | “acupuncture”[Mesh] |
| #12 | “moxibustion” |
| #13 | “electroacupuncture” |
| #14 | “auricular acupuncture” |
| #15 | “warm needling” |
| #16 | “scalp acupuncture” |
| #17 | “acupoint” |
| #18 | “acupoint injection” |
| #19 | “acupoint embedding” |
| #20 | “Intradermal needle” |
| #21 | “Acupoint stimulation” |
| #22 | “transcutaneous electrostimulation” |
| #23 | “Autohemotherapy” |
| #24 | “Cupping” |
| #25 | “Bloodletting” |
| #26 | “Pricking blood therapy” |
| #27 | #11 OR #12 OR #13 OR #14 OR #15 OR #16 OR #17 OR #18 OR #19 OR #20 OR #21 OR #22 OR #23 OR #24 OR #25 OR #26 |
| #28 | #7 AND #10 AND #27 |

| **Supplemental 1.2: Search strategy for Embase** | | |
| --- | --- | --- |
| **Sequence** | **Search term** | **result** |
| #1 | “urticaria” :ab,ti |  |
| #2 | “chronic urticaria” :ab,ti |  |
| #3 | “hives” :ab,ti |  |
| #4 | “nettle-rash” :ab,ti |  |
| #5 | “Angioedema” :ab,ti |  |
| #6 | “Wind cluster” :ab,ti |  |
| #7 | #1 OR #2 OR #3 OR #4 OR #5 OR #6 | 35,724 |
| #8 | “systematic review” :ab,ti |  |
| #9 | “meta analysis” :ab,ti |  |
| #10 | #8 OR #9 | 411,283 |
| #11 | “acupuncture” :ab,ti |  |
| #12 | “moxibustion” :ab,ti |  |
| #13 | “electroacupuncture” :ab,ti |  |
| #14 | “auricular acupuncture” :ab,ti |  |
| #15 | “warm needling” :ab,ti |  |
| #16 | “scalp acupuncture” :ab,ti |  |
| #17 | “acupoint” :ab,ti |  |
| #18 | “acupoint injection” :ab,ti |  |
| #19 | “acupoint embedding” :ab,ti |  |
| #20 | “Intradermal needle” :ab,ti |  |
| #21 | “Acupoint stimulation” :ab,ti |  |
| #22 | “transcutaneous electrostimulation” :ab,ti |  |
| #23 | “Autohemotherapy” :ab,ti |  |
| #24 | “Cupping” :ab,ti |  |
| #25 | “Bloodletting” :ab,ti |  |
| #26 | “Pricking blood therapy” :ab,ti |  |
| #27 | #11 OR #12 OR #13 OR #14 OR #15 OR #16 OR #17 OR #18 OR #19 OR #20 OR #21 OR #22 OR #23 OR #24 OR #25 OR #26 | 46,345 |
| #28 | #7 AND #10 AND #27 | 16 |

| **Supplemental 1.3: Search strategy for Cochrane Library** | | |
| --- | --- | --- |
| **Sequence** | **Search term** | **result** |
| #1 | “acupuncture” :ti,ab,kw |  |
| #2 | “moxibustion” :ti,ab,kw |  |
| #3 | “electroacupuncture” :ti,ab,kw |  |
| #4 | “auricular acupuncture” :ti,ab,kw |  |
| #5 | “warm needling” :ti,ab,kw |  |
| #6 | “scalp acupuncture” :ti,ab,kw |  |
| #7 | “acupoint” :ti,ab,kw |  |
| #8 | “acupoint injection” :ti,ab,kw |  |
| #9 | “acupoint embedding” :ti,ab,kw |  |
| #10 | “Intradermal needle” :ti,ab,kw |  |
| #11 | “Acupoint stimulation” :ti,ab,kw |  |
| #12 | “transcutaneous electrostimulation” :ti,ab,kw |  |
| #13 | “Autohemotherapy” :ti,ab,kw |  |
| #14 | “Cupping” :ti,ab,kw |  |
| #15 | “Bloodletting” :ti,ab,kw |  |
| #16 | “Pricking blood therapy” :ti,ab,kw |  |
| #17 | #1 OR #2 OR #3 OR #4 OR #5 OR #6 OR #7 OR #8 OR #9 OR #10 OR #11 OR #12 OR #13 OR #14 OR #15 OR #16 | 23,778 |
| #18 | “urticaria” :ti,ab,kw |  |
| #19 | “chronic urticaria” :ti,ab,kw |  |
| #20 | “hives” :ti,ab,kw |  |
| #21 | “nettle-rash” :ti,ab,kw |  |
| #22 | “Angioedema” :ti,ab,kw |  |
| #23 | “Wind cluster” :ti,ab,kw |  |
| #24 | #18 OR #19 OR #20 OR #21 OR #22 OR #23 |  |
| #25 | #17 AND #24 | 82 |
| #26 | “systematic review” :ti,ab,kw |  |
| #27 | “meta analysis” :ti,ab,kw |  |
| #28 | #26 OR #27 |  |
| #40 | #17 AND #24 AND #28 | 1 |

| **Supplemental 1.4: Search strategy for Web of science** | |
| --- | --- |
| **Sequence** | **Search term** |
| #1 | Acupuncture OR moxibustion OR electroacupuncture OR auricular acupuncture OR warm needling OR scalp acupuncture OR acupoint OR acupoint injection OR acupoint catgut embedding OR acupoint embedding OR Intradermal needle OR Acupoint stimulation OR transcutaneous electrostimulation OR Autohemotherapy OR Cupping OR Cupping therapy OR Dry cupping OR Wet cupping OR Bloodletting OR Pricking cupping OR Pricking blood therapy |
| #2 | Urticaria OR Chronic urticaria OR hives OR nettle-rash OR Angioedema OR Rubella OR Wind cluster |
| #3 | systematic review OR meta analysis |
| #4 | #1 AND #2 AND #3 |

| **Supplemental 1.5: Search strategy for CNKI** | | |
| --- | --- | --- |
| **Sequence** | **Search term** | **result** |
| #1 | '荨麻疹'+'慢性荨麻疹'+'风团'+'风疹'+'瘾疹'+'血管性水肿', SU | 18,779 |
| #2 | '针刺'+'针灸'+'毫针'+'艾灸'+'温针灸'+'电针'+'耳针'+'体针'+'腹针'+'温针'+'头皮针'+ '浮针'+'腧穴'+'经穴'+'穴位'+'穴位刺激'+'穴位注射'+'穴位埋线'+'皮内针'+'经皮电刺激'+'热敏灸'+'自血'+'自体血清'+'拔罐'+'罐法', SU | 305,908 |
| #3 | '系统评价'+' Meta分析'+'系统综述', FT | 64,927 |
| #4 | #1 AND #2 AND #3 | 100 |
| **Supplemental 1.6: Search strategy for CBM** | | |
| **Sequence** | **Search term** | **result** |
| #1 | "荨麻疹" or "慢性荨麻疹" or "风团" or "风疹" or "瘾疹" or "血管性水肿" | 19,848 |
| #2 | "针灸" or "针刺" or "毫针" or "艾灸" or "温针灸" or "电针" or "耳针" or "体针" or "腹针" or "温针" or "头皮针" or "浮针" or "腧穴" or "经穴" or "穴位" or "穴位刺激" or "穴位注射" or "穴位埋线" or "皮内针" or "经皮电刺激" or "热敏灸" or "自血" or "自体血清" or "罐法" or "拔罐" or "耳穴" | 288,856 |
| #3 | "系统评价" or "meta 分析" or "系统综述" | 51,929 |
| #4 | #1 AND #2 AND #3 | 18 |
| **Supplemental 1.7: Search strategy for Wan Fang** | | |
| **Sequence** | **Search term** | **result** |
| #1 | "荨麻疹" or "慢性荨麻疹" or "风团" or "风疹" or "瘾疹" or "血管性水肿" | 28,370 |
| #2 | "针灸" or "针刺" or "毫针" or "艾灸" or "温针灸" or "电针" or "耳针" or "体针" or "腹针" or "温针" or "头皮针" or "浮针" or "腧穴" or "经穴" or "穴位" or "穴位刺激" or "穴位注射" or "穴位埋线" or "皮内针" or "经皮电刺激" or "热敏灸" or "自血" or "自体血清" or "罐法" or "拔罐" or "耳穴" | 405,296 |
| #3 | "系统评价" or "meta 分析" or "系统综述" | 56,981 |
| #4 | #1 AND #2 AND #3 | 26 |

| **Supplemental 1.8: Search strategy for VIP** | | |
| --- | --- | --- |
| **Sequence** | **Search term** | **result** |
| #1 | U=荨麻疹 OR U=慢性荨麻疹 OR U=风团 OR U=风疹 OR U=瘾疹 OR U=血管性水肿 | 20,198 |
| #2 | U=针刺 OR U=罐法 OR U=拔罐 OR U=针灸 OR U=毫针 OR U=艾灸 OR U=温针灸 OR U=电针 OR U=腹针 OR U=耳针 OR U=耳穴 OR U=体针 OR U=温针 OR U=头皮针 OR U=浮针 OR U=腧穴 OR U=经穴 OR U=穴位 OR U=穴位刺激 OR U=穴位注射 OR U=穴位埋线 OR U=皮内针 OR U=经皮电刺激 OR U=热敏灸 OR U=自血 | 561,669 |
| #3 | R=系统评价 OR R=meta 分析 OR R=系统综述 | 207,997 |
| #4 | #1 AND #2 AND #3 | 15 |
